# Supplementary material for: Identification of photoperiod-induced specific miRNAs in the adrenal glands of Sunite sheep (Ovis aries)
Source: Front Vet Sci. 2022 Jul 22;9:888207. doi: 10.3389/fvets.2022.888207 (PMC9354845; doi:10.3389/fvets.2022.888207)
Supplement: Supplementary file 1 [file Data_Sheet_1.ZIP › Supplementary file/miRDeep2 file.zip!/miRDeep2 file/Novel_Structure/SPLP42A3/Novel_658.pdf]

Provisional ID	:	NC_019484.2_39158
Score total	:	1.9
Score for star read(s)	:	-1.3
Score for read counts	:	0
Score for mfe	:	1.6
Score for randfold	:	1.6
Score for cons. seed	:	
Total read count	:	929
Mature read count	:	929
Loop read count	:	0
Star read count	:	0

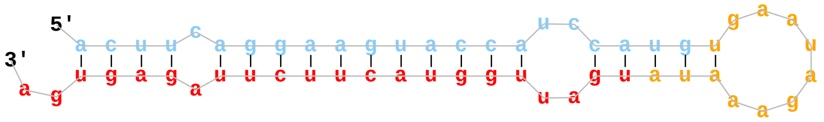

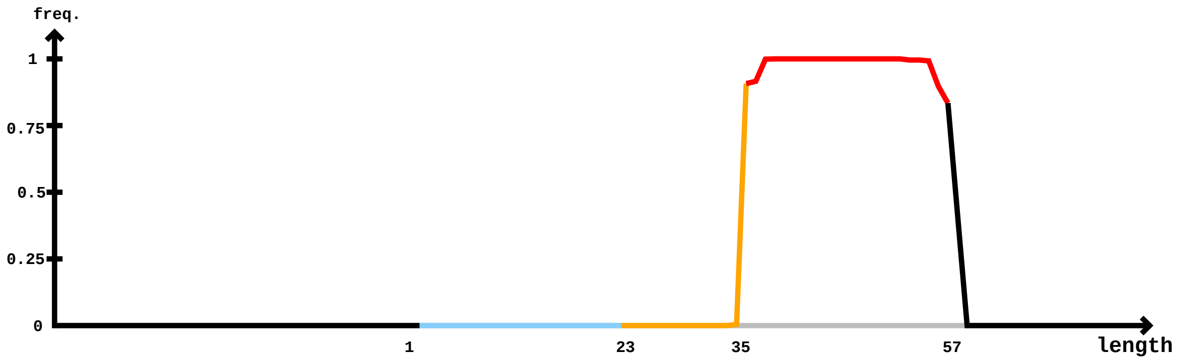


```
                                      Star                            Mature
5'- ggucaccacauuucacuaugccgcgugcaguauugcacuucaggaaguaccauccaugugaauagaaauaugauugguacuucuuagagugaaguaaugcauagcaaauaca   -3'    exp
    (((................)))(((((((.((((.(((((.(((((((((((..(((((........)))))..))))))))))).))))).)))).))))).)).......     reads      mm         sample
    .....................................................................augauugguacuucuuagagu......................     2          0          seq
    .....................................................................augauugguacuucuuagaguga....................     2          0          seq
    ......................................................................ugauugguacuucuuag.........................     4          0          seq
    ......................................................................ugauugguacuucuuagag.......................     3          0          seq
    ......................................................................ugauugguacuucuuagagu......................     86         0          seq
    ......................................................................ugauugguacuucuuagagug.....................     56         0          seq
    ......................................................................ugauugguacuucuuagaguga....................     665        0          seq
    ......................................................................ugauugguacuucuuagagugaa...................     25         0          seq
    .......................................................................gauugguacuucuuagagug.....................     2          0          seq
    .......................................................................gauugguacuucuuagaguga....................     6          0          seq
    ........................................................................auugguacuucuuagagug.....................     1          0          seq
    ........................................................................auugguacuucuuagaguga....................     69         0          seq
    ........................................................................auugguacuucuuagagugaa...................     7          0          seq
    .........................................................................uugguacuucuuagaguga....................     1          0          seq
```
